# Selfish evolution of placental hormones

**DOI:** 10.1093/emph/eoac031

**Published:** 2022-08-19

**Authors:** Grace Keegan, Manus M Patten

**Affiliations:** Department of Biology, Georgetown University, Washington, DC 20057, USA; Department of Biology, Georgetown University, Washington, DC 20057, USA

**Keywords:** gestational drive, selfish genetic element, chorionic gonadotropin, pregnancy, infertility

## Abstract

We hypothesize that some placental hormones—specifically those that arise by tandem duplication of genes for maternal hormones—may behave as gestational drivers, selfish genetic elements that encourage the spontaneous abortion of offspring that fail to inherit them. Such drivers are quite simple to evolve, requiring just three things: a decrease in expression or activity of some essential maternal hormone during pregnancy; a compensatory increase in expression or activity of the homologous hormone by the placenta; and genetic linkage between the two effects. Gestational drive may therefore be a common selection pressure experienced by any of the various hormones of mammalian pregnancy that have arisen by tandem gene duplication. We examine the evolution of chorionic gonadotropin in the human lineage in light of this hypothesis. Finally, we postulate that some of the difficulties of human pregnancy may be a consequence of the action of selfish genes.

## INTRODUCTION

Haig [1] coined the term ‘gestational drive’ to describe a situation in which a gene or haplotype biases maternal investment during pregnancy toward offspring that inherit it or that biases investment away from offspring that fail to inherit it. The term highlights the similarity of their logic to that of traditional meiotic drivers—e.g. spore killers in ascomycete fungi [2] and the Segregation Distorter complex of *Drosophila* [3]—which similarly gain a transmission advantage when found in heterozygous carriers. As is the case with meiotic drivers, gestational drivers can spread in populations despite exacting various fitness costs on their carriers, so long as the costs are offset by gains made through transmission.

In his initial introduction of the concept, Haig [1] pointed to the possibility of ‘green-bearded’ gestational drivers, in which the driving element is expressed by both mother and offspring and recognizes copies of itself—e.g. via a cell-surface adhesion molecule—to effect greater transfers of resources from mothers to offspring. He also considered the possibility for linked signals and receptors, which could function as greenbeards. However, we point to an even simpler mechanism for gestational drive, one that requires no inter-molecular recognition whatsoever.

**Figure 1.**
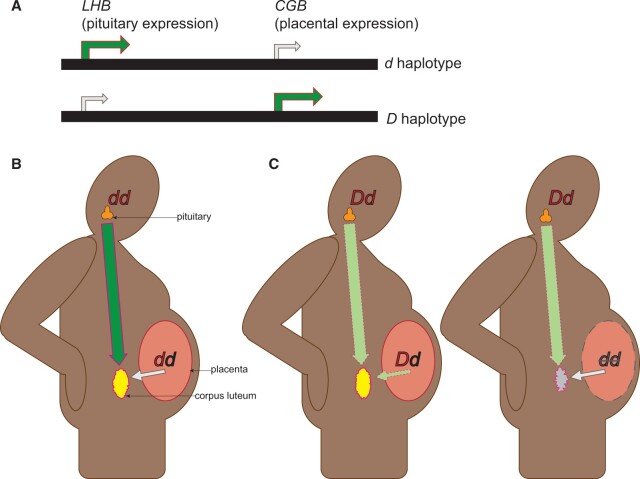
The gestational drive model of evolution at the LHB and CGB genes. (**A**) The two haplotypes differ in two ways. The wildtype, d, shows relatively low expression from the placentally expressed gene (CGB) but high expression from the pituitary-expressed gene (LHB). The driving haplotype, D, combines low expression maternally with high expression placentally. (**B**) Mothers with a dd genotype produce adequate hormone to sustain the corpus luteum through an early stage of pregnancy. (**C**) Mothers with a Dd genotype produce less hormone than their dd counterparts, and only those offspring who inherit D (maternally derived allele written first) are able to compensate for the reduced maternal expression in order to sustain the corpus luteum through early pregnancy. Offspring of Dd mothers who inherit d cannot produce sufficient hormone to sustain the corpus luteum and are spontaneously aborted.

Our gestational driver can be assembled from the simplest of mutations. We consider a gene that is expressed by mothers and that encodes something essential for the maintenance of pregnancy, e.g. a maternal hormone. We then suppose that this gene has duplicated in tandem and has evolved placental expression in one of the paralogs such that there can now be allocrine signaling from the offspring to the mother. With just these features already in place, we have the components for making a selfish genetic element. Haplotypes that combine slightly reduced maternal expression or activity from the first gene with a compensated, increased level of placental expression or activity from the second linked gene will drive at the expense of alternative haplotypes (Fig. 1). Alternatively, one could achieve drive in the absence of a gene duplication. If a single gene is expressed in both mothers and placentae and a single mutation pleiotropically reduces expression in the mother and increases expression in the placenta of offspring who inherit it, then such a mutation would also drive against the wildtype allele. The strategic logic is simple: the driving element makes mothers generally resistant to pregnancy, and only those offspring who inherit the element causing such resistance can withstand it. To our knowledge, this basic mechanism—of delaying the production of some essential product until after fertilization—was first described by Burt and Trivers ([4], p. 55) and has since been implemented by Chen *et al*. [5] in a synthetically designed gene-drive construct in *Drosophila*, but it is otherwise unknown in nature.

These haplotypes exhibit what Haig [6] vividly described as ‘conspiratorial epistasis’. The conspiracy here is to have mothers consistently underprovide something essential for a successful pregnancy and have their offspring—specifically those who inherit the element—provide it instead. Unlike many well-studied drivers, where the conspiracy is for one gene to produce a ‘toxin’ for which the other gene offers an ‘antidote’ or at least an ‘insensitivity’ allele (see Refs. [7, 8] for reviews of this strategy), our gestational drivers require neither a novel, toxic substance nor an antidote. In our drivers, the threat comes from the absence of some essential product—not the presence of a novel harmful substance.

The evolutionary dynamics of this sort of gestational driver are quite similar to those of the Medea element of flour beetles, which, like our gestational driver, causes the elimination of offspring that fail to inherit the driving haplotype from their heterozygous mothers (Box 1). The population genetic details of Medea have been worked out previously [9, 10, 11]. A few of the results of this earlier theoretical work are noteworthy. The first is that these elements, much like typical meiotic drive elements, can spread in populations despite exacting various costs on their bearers. One contrast between gestational and typical meiotic drivers, though, is that gestational drivers have more opportunity for polymorphism. Their spread is hindered at higher frequencies because paternal transmission of the driving haplotype, which becomes more common at high frequencies, prevents heterozygous mothers from killing offspring that inherit their non-driving haplotype. Polymorphism is also made more likely if the driving allele confers costs on mothers—i.e. if their lifetime reproductive success is reduced by virtue of carrying one or two copies of the haplotype, or in other words, if the loss of offspring who do not inherit the haplotype is not compensated for by the production or survival of additional offspring who do inherit it [9, 10] (Ward et al. 2010). In the absence of fecundity or fertility effects, driving haplotypes are expected to reach fixation. Fixation can also happen when heterozygotes have lower fitness than either homozygote (Ward et al. 2010).

Box 1 The population genetics of gestational drive

Although the mechanism we discuss in the main text involves two genes (one for a maternally expressed product and the other for a placentally expressed product), it is simpler to think of this pair of genes as a single locus, a reasonable approximation when the recombination frequency between the two genes is low. Our single locus has two alleles: a driving allele, D and a non-driving, wildtype allele, d. Mothers that are heterozygous are altered by the presence of D in such a way that any of their offspring who inherit d as opposed to D are disadvantaged in early development (unless they inherit a D allele from their father). At the extreme, offspring of Dd mothers that fail to inherit a D allele from either parent are spontaneously aborted.

We can capture this and clarify the evolutionary logic of gestational drive with a simple population genetic model. (We make no attempt here to capture every possible complexity of such a system and refer the reader to earlier treatments of Medea’s dynamics by Wade and Beeman [10] and Ward et al. [11] for thorough analyses.) In the pregnancies of Dd mothers, dd offspring will survive with a probability relative to their D-bearing siblings equal to 1–*t*, with 0 ≤ *t* ≤ 1. To derive the invasion condition of a novel D allele in an otherwise d population, we compare the average fitness of D and d alleles near the boundary where d is fixed. The relevant comparison is between the success of a single d allele in a dd mother to that of a single D allele in a rare Dd mother. Let’s assume that dd mothers produce in their lifetime *N* viable offspring and that Dd mothers produce an amount equal to (1–*s*)*N*. These values take account of both fecundity differences among mothers and fertility differences (with fertility here calculated when mated to the most common dd males). The d allele of a dd mother will find itself in ½*N* offspring and the D allele of a Dd mother will find itself in (1/(2–*t*))(1–*s*)*N* offspring. The condition for spread is *t* > 2 *s*, showing how the driving allele can spread in a population provided its killing effects outweigh any losses to maternal fertility. Reproductive compensation, where a lost pregnancy can be followed in short order by another pregnancy, will tend to keep s small and facilitate the invasion of a driving allele. For fixation, a similar analysis can be done, comparing a rare d allele with the now common D allele, assuming that all offspring of DD mothers are viable and that the Dd offspring of Dd mothers are as viable as their DD siblings. This will show that the driver will fix provided DD mothers produce more viable offspring (i.e. >(1–*s*)*N*) than Dd mothers.

In population genetic models of Medea, it is typically assumed that fecundity and fertility will be lowest in DD mothers, reflecting a cost of harboring a toxic D allele. This thwarts their spread and makes polymorphism more likely. But with gestational drive, where D represents a different strategy for killing that does not involve the production of a toxin, it is reasonable to suppose that Dd mothers have the lowest fertility, ranking below even DD mothers, owing to the spontaneous abortions Dd mothers experience. So long as DD mothers have higher fertility than Dd mothers, one expects D to fix. Thus a driver that harms both Dd and DD mothers, but harms Dd mothers more, will fix, and this would entail the evolution of reduced fertility.

Once a driving element reaches fixation, the hormonal control for maintaining pregnancy will have shifted, at least in part, from mothers to their offspring. We want to stress that such an evolutionary change—from maternal to offspring control of pregnancy maintenance—need not provide any benefits to mothers nor offspring. The adaptive benefit of a gestational driver can be entirely to the gene or genes responsible.

## hLH AND hCG: A HISTORY OF GESTATIONAL DRIVE?

We can further examine the gestational drive hypothesis by turning our attention to a specific example. We have chosen luteinizing hormone and chorionic gonadotropin to illustrate how a gestational drive mechanism might work and to show what tests are possible.

Maintaining early pregnancy in mammals is a matter of prolonging the lifespan of the corpus luteum [12], which is the major source of progesterone in early pregnancy. Despite this common aim, the details of early pregnancy and the signaling involved in maintaining the corpus luteum are varied in mammals. In humans specifically, luteinizing hormone (hLH), which is secreted by the maternal pituitary in response to stimulation by gonadotropin-releasing hormone (GnRH), is essential for maintaining the corpus luteum during the luteal phase of a mother’s menstrual cycle [13] but then sees its production fall off prior to luteal regression [14]. In pregnancy, a second luteotrophic hormone, human chorionic gonadotropin (hCG), is detectable from human embryos as a transcript as early as the two-cell stage [15] and can be detected in maternal blood as early as 6 days post-fertilization [16]. The placentally provided hCG ‘rescues’ the corpus luteum in early pregnancy. Until the luteoplacental shift—at which point the placenta can produce sufficient progesterone on its own—the corpus luteum, sustained by hCG, is essential for the continuation of pregnancy.

Luteinizing hormone (LH) and chorionic gonadotropin (CG) are glycoprotein hormones that comprise a common α subunit, encoded by a gene on human chromosome 6, but that differ in their β subunits, which are produced from duplicate genes that reside in tandem on human chromosome 19 [17]. These paralogs arose 35-55 million years ago in the lineage leading to anthropoid primates (i.e. after the split between monkeys and tarsiers but before the split between new- and old-world monkeys [18]). Further evolution has ensued in this lineage: e.g. new world monkeys subsequently pseudogenized their original LHB gene and now use CGB for both CG and LH [19]; copy number of CGB varies widely across the old-world monkeys and apes; and bouts of seemingly adaptive evolution at the CGB gene or genes are scattered across the branches of the primate phylogeny [18].

Our speculation for the course of events in the history of the human LH/CG system runs as follows. In an ancestral primate, pituitary LH secretion or action was sufficient on its own to prolong the survival of the corpus luteum and prepare the uterus for the embryo’s implantation for some time. A duplicated version of the β subunit gene then arose and spread to fixation—either owing to a selective advantage or simply by chance. This duplicate gene then evolved placental expression, giving rise to a bona fide CG hormone. We posit that CGB expression levels varied in the population owing to allelic variation at the CGB locus, as is currently seen in the human population [20]. A mutation to LHB that reduced the duration or amount of its expression following ovulation and in the earliest stages of pregnancy arose on a haplotype with high expression of CGB, thus producing a driving element. Mothers who were heterozygous for the element were, all things equal, poised to reject pregnancies in the earliest stages owing to the regression of their corpus luteum unless the offspring inherited the conspiring haplotype that enabled its rescue through elevated production of CG from trophoblast (Fig. 1). This driving haplotype spread and reached fixation, thus shortening—or in some other way modifying—the luteal phase of the ovulatory cycle. Later mutations to LHB and CGB with similar effects could have subsequently arisen, driven and reached fixation.

Across primate lineages the mutations that achieve these effects may be different. For example, the allelic variation at CGB might reflect cis-regulatory variation or copy number variation, such that the quantity of CG is affected, or it might reflect coding variation that affects the quality of the protein to change its biological half-life. Although the outcomes may differ in detail, they share the property that offspring evolve a greater responsibility for the maintenance of early pregnancy and mothers become increasingly resistant to it.

In the above version of events, gestational drive is not meant to explain the origin of CG from LH, merely the subsequent evolution of the two hormones after CG has already originated. However, a different ordering of the events could see a role for gestational drive in the actual origin of the CG hormone. Henke and Gromoll [17] suggest that the gene duplication at LHB initially gave rise to a redundant copy of LHB—i.e. one that also manufactured LH from the pituitary. We may then suppose that expression levels of LHB were subsequently modified to accommodate the extra gene dose. Then we suppose that a cis-regulatory mutation to one of the copies caused restriction of its expression to the placenta and left the other copy unaffected. This haplotype would have displayed the conspiratorial epistasis described above: it would have paired insufficient production of a hormone by mothers with a rescuing dose from placenta. This version of events also requires the prior evolution of placental expression of the alpha subunit at some unspecified point, which admittedly makes for an unlikely scenario for CG’s origin. But, at the very least, this exercise demonstrates how such selection pressure can in principle operate at the origin of novel placental hormones.

We want to stress that the mutations that effect such strategies in all the scenarios described above must be linked. For example, a mutation that reduced the expression of GnRH, which acts in the pituitary to stimulate production of LH, would not be a driving mutation even though it achieves the same phenotype as the LHB mutations mentioned above. Because it would be unlinked to the compensating mutation at CGB—GnRH is on human chromosome 8—it would be unable to enhance its own transmission. Likewise, a mutation that rendered the LH receptor less sensitive to LH/CG would also make a mother resistant to pregnancy, but such a mutation cannot drive. The receptor gene, on human chromosome 2, is also unlinked to CGB. Any evolution of GnRH or the receptor would need to be attributed to a different evolutionary process. (Below we discuss McCoy and Haig’s [21] parent-offspring conflict hypothesis for CG’s evolution, which does apply to the evolution of unlinked genes.)

There is a variant LHB gene that occurs at a frequency between 7% and 42% in various Northern hemisphere countries [22, 23]. The variant has a shorter half-life *in vivo* and shows lower bioactivity [23] and has been associated with recurrent spontaneous abortion, a short luteal phase, and reduced fertility [24–26]. Themmen and Huhtaniemi [27] suggest that this variant provided some yet unknown benefit to females in an ancestral population, and thus natural selection explains its current prevalence. Another explanation, rooted in gestational drive, is that this variant, if paired on a haplotype with a CGB allele that can compensate for its low performance, perhaps by having a longer half-life itself, could have spread by drive through the population to its current relatively high frequency. It would be interesting to measure the transmission ratio of this haplotype in offspring born to heterozygous mothers and to seek differences in the quality or quantity of CGB produced by it.

## PREDICTIONS OF THE GESTATIONAL DRIVE HYPOTHESIS

Considering hormones produced by placentae that act systemically in pregnant mothers, rather than locally near the site of implantation, we expect gestational drivers to be found more commonly in species that birth singletons rather than multiples. The benefit of drive to the conspiring haplotype is difficult to collect in species that gestate multiple offspring at once. Placental hormones that act systemically in pregnant mothers become something like a public good to the members of a litter [28]. A litter born to a heterozygous mother should comprise an even mix of her two haplotypes at the drive locus. This creates two possible hurdles for the conspiring haplotype. First, it is possible that the half of offspring who inherit the rescuing allele at the placentally expressed locus aren’t able collectively to produce enough hormone to sustain the pregnancy, and so the sabotaging allele at the maternally expressed locus ends up harming itself in the end. Second, if instead the half of the litter that produces the rescuing effect is able to sustain pregnancy, then that sustenance is likely to benefit all offspring equally rather than just the conspirators. Either way, with litters of multiples the conspiring haplotype cannot easily monopolize pregnancies the way it can in species that carry singletons. One pattern that would be consistent with—though by no means solid proof of—the gestational drive hypothesis, is that reproductive inefficiency should be higher in species with singleton births rather than litters, owing to a greater opportunity for the invasion of gestational drivers.

There are multiple ways to maintain a corpus luteum. LH and CG have luteotrophic properties, but there are also anti-luteolytic factors that would likewise contribute to prolonging the corpus luteum’s lifespan [12]. As a lineage evolves from multiples to singletons, mutations that target either manner of sustaining the corpus luteum can generate gestational drivers, and novel drivers could end up becoming what appear to be novel signals of early pregnancy. In ruminants, the signal of early pregnancy is interferon-τ, which is unique to their lineage and is encoded by the IFNT gene, a tandem duplicate of the gene for IFN-ω (IFNW) [29, 30]. IFN-τ serves an anti-luteolytic function by blocking the action of prostaglandin F2α, which itself causes regression of the corpus luteum. Little is known about the role of IFNW in early pregnancy, but a prediction of the gestational drive model would be that it shows maternal expression and contributes to the maintenance of early pregnancy in the closest relatives of the ruminants that lack an IFNT gene. Further, on the gestational drive hypothesis, one would predict that IFNW had shed this role evolutionarily in the ruminants since IFNT gained expression in trophoblast.

An evolutionary pattern like the above, in which mothers retreat from producing some hormone and a placental ortholog steps in to take over its production, is a key prediction of the gestational drive hypothesis. We see something just like this in the production of prolactin and placental lactogens in muroid rodent pregnancies, at least on a developmental timescale. The prolactin gene has duplicated repeatedly in the rodent lineage and acquired placental expression to give rise to placental lactogens and additional placental hormones [31]. In these rodents, maternal production of prolactin from the pituitary declines just as production of placental lactogens increases (see Figure 4 of Ref. [31]). These hormones have systemic luteotrophic effects, though if drive is at play, it seems more likely that one of the more local effects of placental lactogens—e.g. on angiogenesis near the site of implantation—would be involved.

## RELATIONSHIP TO PARENT-OFFSPRING CONFLICT THEORY

Haig [32] and McCoy and Haig [21] offer a different, though non-mutually exclusive, view of CG’s evolution that rests on the theory of parent-offspring conflict [33]. In their explanation, mothers are selected to examine early embryos for quality—in effect, to estimate the expected inclusive fitness return on their parental investment—and the fetus’ ability to produce CG serves as a proxy of its quality. Over time, mothers have evolved an increasingly difficult exam for offspring to pass. Mothers can accomplish this evolutionarily by ratcheting down their contribution to sustaining the pregnancy or by being further insensitive to the action of fetal hormones. If only high-quality offspring are able to mount a sufficient CG signal and pass the test, then choosy mothers can ensure that their offspring provide an inclusive fitness return on their investment. In response, offspring have evolved—via modification of the quality or quantity of CG—to pass the maternal test, quite possibly in ways that game the system. For example, offspring can produce a stronger signal at no cost by evolving a mutated version of CG with an increased half-life. This sets up a co-evolutionary dynamic, with mothers evolving to become more and more resistant to CG’s effects, and therefore more resistant to pregnancy, and offspring evolving to express greater levels of CG to increase the likelihood of their being retained through early pregnancy.

The canonical pattern predicted by gestational drive, in which maternal expression of a product gives way evolutionarily to placental expression of that same product to sustain pregnancy, is also predicted by parent-offspring conflict theory. Whenever offspring favor more of some hormone in maternal circulation than mothers do, one expects in the evolutionary long-run to find that mothers cease producing that product and offspring take full control, a result that Haig [34] calls the ‘loudest voice prevails principle’. We thus have two different ways of explaining the same observation. Ideally, there would be a way to judge which explanation applies. We think the solution lies in measuring the fitness effects of early pregnancy losses.

Under the parent-offspring conflict theory, the high rate of early pregnancy loss in humans—as many as 40–50% of fertilized ova fail to implant [35]—might be viewed in one of two ways. First, it may be seen as just a maladaptive byproduct of conflict. When selection works at cross purposes, it can leave the bearers of its designs worse off. In this case, some modest loss of pregnancies, of those with the lowest expected return on investment—say, the bottom 20%—might be adaptive for mothers, but we end up with a system that, owing to escalation between the two parties, is imperfectly designed and fraught (e.g. Ref. [36]), leading to an excessive loss of pregnancies. Alternatively, what we might label reproductive ‘inefficiency’ might well be the signature of a system working effectively to screen out low-viability embryos; it might simply be that only ∼50% of embryos would ever recoup the investment that a human mother would place in them. A point in favor of this view is that a large fraction of spontaneously aborted embryos appear to have chromosomal abnormalities. That said, a large fraction of embryos overall show chromosomal instability [37], and whether within-embryo selection for euploidy is sufficient to overcome this challenge remains an open question. In either case, this hypothesis permits an interpretation that, at least to some extent, the extensive reproductive inefficiency of human pregnancy is adaptive and serves the fitness interests of mothers. The gestational drive hypothesis, on the other hand, finds no direct fitness benefit of these pregnancy losses for mothers nor the aborted offspring. The benefits are to the driving haplotype itself. A considerable loss of early pregnancies is precisely what one would expect if gestational drivers are at work in populations. For a gestational driver, the loss of a pregnancy is neither collateral damage nor side effect; it is the intended effect. Whether the amount of early pregnancy loss exceeds that which maximizes maternal fitness is the essential—albeit quite difficult—empirical question.

It is possible, of course, that these two ways of explaining the patterns are at work simultaneously. A placental hormone may arise by gestational drive and then see its expression level and the maternal receptors for it evolve subject to parent-offspring conflict. Likewise, placental hormones may originate for some purpose altogether unrelated to parent-offspring conflict or gestational drive, but then be shaped by recurring bouts of parent-offspring conflict and gestational drive. We do not offer the gestational drive hypothesis as a complete alternative to parent-offspring conflict. Our interests here are simply to draw attention to the special evolutionary dynamics that are seemingly unavoidable for linked pairs of genes and the harm they may bring to reproductive health.

## Data Availability

No new data were generated or analysed in support of this research.
